# Participation in organised sport to improve and prevent adverse developmental trajectories of at‐risk youth: A systematic review

**DOI:** 10.1002/cl2.1381

**Published:** 2024-01-17

**Authors:** Trine Filges, Mette Verner, Else Ladekjær, Elizabeth Bengtsen

**Affiliations:** ^1^ VIVE – The Danish Centre of Applied Social Science Copenhagen Denmark; ^2^ VIVE – The Danish Centre of Applied Social Science Aarhus Denmark; ^3^ Steno Diabetes Center Aarhus Denmark

## Abstract

**Background:**

Healthy after‐school activities such as participation in organised sport have been shown to serve as important resources for reducing school failure and other problem/high‐risk behaviour. It remains to be established to what extent organised sport participation has positive impacts on young people in unstable life circumstances.

**Objectives:**

What are the effects of organised sport on risk behaviour, personal, emotional and social skills of young people, who either have experienced or are at‐risk of experiencing an adverse outcome?

**Search Methods:**

The database searches were carried out in March 2023 and other sources were searched in May 2023. We searched to identify both published and unpublished literature.

**Selection Criteria:**

The intervention was participation in leisure time organised sport. Young people between 6 and 18 years of age, who either have experienced or are at‐risk of experiencing an adverse outcome were eligible. Primary outcomes were problem/high‐risk behaviour and a secondary outcomes social and emotional outcomes. Studies that used a control group were eligible for. Studies that utilised qualitative approaches were not.

**Data Collection and Analysis:**

The number of potentially relevant studies was 43,716. Thirteen studies met the inclusion criteria. Only seven studies could be used in the data synthesis. Five studies were judged to have a critical risk of bias and were excluded from the meta‐analysis. One study did not report data that enabled the calculation of effect sizes and standard errors. Meta‐analyses were conducted on each conceptual outcome separately. All analyses were inverse variance weighted using random effects statistical models.

**Main Results:**

Two studies were from Canada, one from Australia, and the remaining from the USA. The timespan of the interventions was 23 years, from 1995 to 2018. The median number of participants analysed was 316, and the median number of controls was 452. A number of primary outcomes were reported but each in a single study only. Concerning secondary outcomes, two studies reported the effect on overall psychosocial adjustment at post‐intervention. The standardised mean difference was 0.70 (95% CI 0.28–1.11). There was a small amount of heterogeneity. Three studies reported on depressive symptoms at 0–3 years follow‐up. The standardised mean difference was 0.02 (95% CI −0.01 to 0.06). There was no heterogeneity between the three studies. In addition, a number of other secondary outcomes were reported each in a single study only.

**Authors' Conclusions:**

There were too few studies included in the meta‐analyses in order for us to draw any conclusion. The dominance of Northern America clearly limiting the generalisability of the findings. The majority of the studies were not considered to be of overall high quality and the process of excluding studies with critical risk of bias from the meta‐analysis applied in this review left us with only 7 of a total of 13 possible studies to synthesise. Further, because too few studies reported results on the same type of outcome, at most three studies could be combined in a particular meta‐analysis and no meta‐analysis could be performed on any of the primary outcomes.

## 1 PLAIN LANGUAGE SUMMARY

### 1.1 Evidence of the effects of organised leisure time sport for at‐risk young people is inconclusive

We aimed to find evidence of the effectiveness of participation in organised leisure time sport on improving risk behaviour, personal, emotional and social skills of young people at risk of an adverse outcome. The evidence is inconclusive because of the small number of studies each reporting different outcomes.

### 1.2 What is this review about?

The intervention is participation in leisure time organised sport. Young people between 6 and 18 years of age, who either have experienced or are at‐risk of experiencing an adverse outcome were eligible. Our primary outcome is problem/risk behaviour and secondary outcomes are social and emotional outcomes.

### 1.3 What is the aim of this review?

We examine the effects of participation in leisure time organised sport on risk behaviour, personal, emotional and social skills of young people compared to no participation in leisure time organised sport.

### 1.4 What studies are included?

Thirteen studies were identified. Only seven were assessed to be of sufficient methodological quality to be included in the final data synthesis. The studies were from Australia, the USA and Canada and spanned the period 1995 to 2018. There were no randomised controlled trials. The studies contained data for 17,155 participants and 9664 controls.

Studies had to examine the impact of participation in leisure time organised sport on at‐risk youth using a comparison group.

### 1.5 What is the effect of organised leisure time sport?

The evidence is inconclusive. A number of primary outcomes were reported but each in a single study only. For secondary outcomes, two studies reported the effect of organised sport participation on overall psychosocial adjustment post‐intervention and three studies reported on depressive symptoms at 0–3 years follow‐up. A number of other secondary outcomes were reported in a single study only.

### 1.6 What do the findings of this review mean?

The impact of participation in leisure time organised sport on at‐risk youth shows has yet to be evaluated thoroughly. The evidence was inconclusive because too few studies reported results on the same type of outcome.

The vast majority of the available evidence used in the data synthesis was from the USA and Canada. The findings may not be generalisable to other settings and systems outside Northern America.

Because too few studies reported results on the same type of outcome, at most three studies could be combined in a particular meta‐analysis. No meta‐analysis could be performed on any of the primary outcomes.

There is a need for more rigorous studies reporting a larger number of outcomes, for example, substance use, delinquency, school suspension and drop out. There is a need for studies conducted in countries outside of Northern America.

### 1.7 How up‐to‐date is this review?

We searched for studies up to 2023.

## 2 BACKGROUND

### 2.1 The problem, condition or issue

Children and adolescents in the United States and in Europe spend more than half of their waking hours on leisure activities (Gracia et al., 2020; Larson & Verma, 1999; Wight et al., 2009). In the US, calculations from the 2003 to 2005 American Time Use Survey showed that youth aged 15–17 on average had 488 min (approximately 8 h) of leisure time per day, net of time spent in school, eating, grooming and doing household work (Wight et al., 2009). The corresponding numbers, using data from the time use surveys from Finland (2009–2010), Spain (2009–2010) and the United Kingdom (2014–2015) were 571 min (approximately 9.5 h) for youth aged 10–17 (Gracia et al., 2020). For many, much of this time is spent either in unstructured, peer‐focused activities or in front of the television, computer, and so forth. For youth aged 15–17 in the US, Wight et al. (2009) report that on average 57 percent of the 488 min of leisure time is spent this way. The corresponding number for European youth aged 10–17 is 50 percent of the time spent on computing programming, Internet use, computer games, watching TV, video watching and unstructured activities (Gracia et al., 2020). Some of this leisure time could probably be spent better in ways that would both facilitate positive development and prevent the emergence of developmental problems (see Eccles & Gootman, 2002).

Leisure time activities such as organised sport are a good option as they provide young people with a valued place within a structured peer‐involved activity. In addition, sport is a voluntary activity that is both intrinsically and extrinsically motivating, and one that links young people to coaches who are positioned to assume the role of caring adult mentors. (Cronin & Allen, 2015; Petitpas et al., 2004) which in particular at‐risk youth may be in need of.

At‐risk youth may be defined as a diverse group of young, socially vulnerable people in unstable life circumstances, who are currently experiencing or are at risk of developing one or more serious problems such as school failure or drop‐out, mental health disorders, substance and/or alcohol abuse, unemployment, long‐term poverty, delinquency and more serious criminal behaviour (Arbreton et al., 2005; Quinn, 1999).

At‐risk youth often come from socio‐economically less advantaged and dysfunctional families (Treskon, 2016). At‐risk youth have often experienced at number of adverse events such as poverty, emotional or physical abuse and neglect, out‐of‐home placement, living with mentally ill or substance‐abusing parents and unstable housing situations. Thus, at‐risk youth often lack stable attachment figures and suitable adult role models.

Participation in organised sport has been shown to serve as an important resource for reducing school failure and other problem/high‐risk behaviour (Parker, 2011). Larson (2000) argued that youth report more positive psychological states in voluntary structured activities, such as sports, because they experience challenge and perceive themselves to be active, in control, and competent in these settings. In addition, compared with other types of leisure activities, Larson et al. (2006) found that youth participating in organised sport reported significantly more experiences related to initiative, emotional regulation, and teamwork.

Also, research by, for example, Camiré et al. (2009); Holt et al. (2009) and Holt and Neely (2011) has revealed relationships between involvement in sport and several positive outcomes for youths' physical, cognitive, psychological, and social development. Additional examples include the ability to cope with pressure, communicate, receive feedback, set goals, solve problems, and deal with successes and failures (Papacharisis et al., 2005).

An example of an intervention based upon empowering is a Danish project engaging children aged 8–15 years old who have psychosocial challenges. The Danish NGO GAME initiated the project in 2018. The children were engaged in parkour activities in four cities in Denmark (Hansen, 2021). The intervention consists of 1 h of training for a period of 32 weeks. The training‐concept focusses upon non‐competitiveness, social pedagogical principles, motivation, manageability and a recurring structure to insure success for all participants. After this, a period of 8 weeks focusing on bridging barriers for the participants to participate in organised sports. The result shows that the participants gain motivation and act on this motivation for being physically active during their leisure time. One‐third of the participants became a lasting member of a sports organisation. Almost half of the participants also experienced gaining new friends (47%), and they strengthened their personal and social competences (Hansen, 2021).

Thus, while the benefits of youth sport participation have been of interest to sport researchers for some time and several systematic reviews are published, no research in the form of a systematic review with meta‐analysis to date has examined the benefits of organised sport for at‐risk youth in particular. It thus remains to be established to what extent organised sport participation have positive impacts on at‐risk youth.

A major difficulty in estimating the causal effects of organised sport participation is the potential endogeneity of the young individual's life circumstance and developmental state that leads to the decision to participate in organised sport. It is thus very important to take self‐selection characteristics into account, or to quote Fullinwider (2006): ‘successfully control variables statistically to cut through the fog of correlation’ (p. 15).

Studies that simply assess the association between organised sports participation and developmental trajectories cannot be used to support conclusions about causation, because these studies are not able to factor out selection effects and there is as little basis for denying the positive contribution of organised sports participation as there is for affirming it.

Hence, considering the fact that the population under investigation in this review by nature volunteer into the intervention, we believe it is vital that an appropriate comparison group and access to relevant pre‐tests is used to establish causality. Studies that control for important confounding factors provide some evidence for considering possible causal effects. While conclusions about causal effects must be very tentative, it is important to extract and summarise the best evidence available.

### 2.2 The intervention

The intervention of interest is organised sport. We will use the following definition of organised sport: a structured activity through an organisation, requiring physical exertion and/or physical skill and is generally accepted as being a sport (e.g., football, hockey, badminton, tennis, etc.). The common meaning of the term ‘sport’ is very wide and includes more disciplines than, for example, the Olympic definition. In Olympic terminology, ‘sport’ refers to all events sanctioned by an international sports federation, and may comprise several disciplines of which not all are necessary Olympic disciplines (the list is constantly changing). Likewise, not all international sports federations are part of the Olympic programme but are members of the General Association of International Sports Federations (GAISF) and are contested at the World Games. Currently, there are 97 member‐Federations where several include multiple disciplines (see https://gaisf.sport/members/).

By its nature, organised sport is competitive, requiring the participants to develop personal discipline, set goals, and strive to reach them and learn to sacrifice for delayed benefits. Generally, there is a coach involved, from which participants follow directions and execute the skills taught. There are certain rules of engagement (regular participation, a certain number of days or hours of practice per week) which, of course, vary by sport, which can be individual or team oriented, contact sport, limited contact or no contact sport, and require different skills and competencies to perform effectively (strength, speed, dexterity, teamwork). The eligible setting is after‐school sport participation, that is, leisure activities, where social skills can be acquired through organised sport participation because of the unique demands of team sports such as the naturally afforded opportunities for youth to display skills such as co‐operation, compromise, teamwork, and leadership. But even participating in individual‐oriented sport, learning is an unavoidable part of the social life and participation in practice required when joining a sports club.

Sport clubs may be school or non‐school sports clubs as long as the activity takes place after school hours. In most European countries, the main means of promotion of sport are non‐school sports clubs, while in the USA (and to some extent Canada) school‐based sport clubs are the main providers of sport (Camiré, 2014; Laios, 1995). The popularity of non‐school sports clubs has, however, increased in the USA during the past couple of decades (Bennett et al., 2020).

### 2.3 How the intervention might work

Participation in organised sport has been shown to serve as an important resource for reducing school failure and other problem/high‐risk behaviour (Parker, 2011). Youth report that they experience challenge and perceive themselves to be active, in control, and competent in these settings and, compared with other types of leisure activities, report significantly more experiences related to initiative, emotional regulation, and teamwork (Larson, 2000; Larson et al., 2006).

There have been several strands of theories offered as a potential understanding of the theory of change behind sport participation (Holt et al., 2017; Jones et al., 2017).

For example, self‐determination theory (SDT) with its focus on the social‐contextual ingredients required for optimal growth and development (Ryan & Deci, 2000) is particularly useful when studying disadvantaged youth and has been extensively used as the theoretical framework guiding sports research (Jones et al., 2017).

SDT is a meta theory of human motivation and personality that addresses autonomous behaviours and conditions and processes that support voluntary engagement. Optimal functioning, development, and well‐being is achieved through the satisfaction of three innate psychological needs, namely autonomy, competence, and relatedness (Ryan & Deci, 2000). SDT is based on the assumption that a person's development, growth and well‐being are supported to the extent that these basic needs are accommodated by the social context. Autonomy is the desire to engage in activities of one's own choosing and the satisfaction of this need involves the experience of choice and the feeling that one is the initiator of one's own actions. Competence reflects the need to have an effect on the environment and to achieve desired outcomes and is fulfilled by the experience that one can effectively bring about desired effects and outcomes. Relatedness refers to the desire to feel securely connected to, understood and valued by others (Ryan & Deci, 2000).

Studies conducted in the sport setting have provided support for these basic tenets of SDT. With respect to the relationship of autonomy support (i.e., the coach is perceived as autonomy supportive by the athletes) to need satisfaction, research has shown that in the context of physical education, perceptions of an autonomy‐supportive climate were strong positive predictors of students’ perceptions of autonomy (Standage et al., 2003). In the same vein, Ryan and Solky (1996) argue that social support may have positive psychological effects if the social support system satisfy one or more of the basic psychological needs SDT is built on, the need for relatedness in particular. The social support system in sports or the athletes' perceptions of social support in their team and by their coach may satisfy the need for relatedness. Research has shown that the team atmosphere created mainly by the coach has a strong influence on the social reality of athletes (Roberts & Treasure, 1992).

Regarding the need for competence, a dimension of the sports environment which in particular may satisfy this need is the coach's emphasis on athletes' self‐referent improvement, mastery, and effort. A mastery of environmental focus of the coach fosters perceptions of competence, because the self‐referenced criteria (e.g., effort) underlying competence judgements and ensuing feelings of success are more controllable and achievable compared to normative criteria (e.g., winning), according to Duda (2001).

Finally, the study by Reinboth et al. (2004), tested and found support for SDT's basic needs in the context of sport. The authors suggest that a social environment which is autonomy supportive, emphasises improvement and effort, and is socially supportive, may help maximise the satisfaction of sport participants’ basic needs, which in turn may foster eudaimonic well‐being (well‐being achieved through experiences of meaning and purpose).

Another strand of theory which offers a potential understanding of the theory of change behind participation in organised sport is *Situated Learning* (Lave & Wenger, 1991). The term ‘situated learning’ refers to learning that occurs within a particular and authentic context through the individual's social participation. Rather than focusing on learning as a primarily cognitive process involving a number of tasks, situated learning theorists study the process in which individuals become new members of a learning community.

In their often cited work: ‘Situated Learning: Legitimate Peripheral Participation’, Lave and Wenger (1991), focus on acquisition of skills and knowledge that takes place outside traditional schooling within communities of practice. Lave and Wenger propose that learning should not be viewed as the mere transmission of knowledge but as a distinctly embedded and active process. Learning is perceived as a contextualised process in which content is learned through doing activities. Furthermore, Lave and Wenger suggest that motivation too is ‘situated’, as learners are naturally motivated by their growing value of participation (Lave & Wenger, 1991). Based on this approach, children and youth participating in organised sport inherently become motivated to learn as this enables them to move from being novices to becoming full participants within the learning community. Reporting on a 3‐month ethnographic study conducted in a swimming club, Light (2010), explored the range of social, personal, and cultural development that occurs through children's participation in the practices of the club, drawing on Lave and Wenger's analytic concepts of situated learning and communities of practice. Light (2010), suggests that a range of important social learning, enculturation, and the development of identity arise from participation in the practices of the swimming club.

### 2.4 Why it is important to do this review

Although participation in organised youth leisure activities such as sport, has been shown to be associated with positive outcomes on general developmental indicators, such as school completion, employment and youth crime (Eccles et al., 2003; Parker, 2011), it is questionable whether the youth who would benefit most are those who chose to attend such programmes (Arbreton & McClanahan, 2002) or are given the opportunity to attend. It has been noted that the availability of such programmes is inequitably distributed across communities – with much lower availability in precisely those communities where the adolescents are at highest risk for poor developmental outcomes (Eime et al., 2015; Fullinwider, 2006; Owen et al., 2022). If there is limited or poor availability of quality facilities and activities in the local neighbourhood, transportation issues may be a barrier to attending organised sport.

And even if programmes are available, they are typically not for free but come with a participation fee and equipment costs out of reach for children living in poverty (Owen et al., 2022). According to Owen (2022) children and adolescents living in higher socioeconomic status households are 1.87 times more likely to participate in sport.

There is a need for strategies to increase the provision of sport opportunities, both facilities and affordability, in childhood and adolescence, to help develop and strengthen children and youths' physical, cognitive, psychological, and social development through sport participation.

To the best of our knowledge, there are currently no systematic reviews assessing what is known about the causal effects of sport participation on at‐risk youth.

## 3 OBJECTIVES

The main objective of this review is to answer the research question: What are the effects of organised sport on risk behaviour, personal, emotional and social skills of young people, who either have experienced or are at‐risk of experiencing an adverse outcome? Further, the review will attempt to answer if the effects differ between participants' characteristics such as gender, age and risk indicator or between types of sport (e.g., team/individual, contact/non‐contact, intensity and duration).

## 4 METHODS

The systematic review protocol (Filges, 2023) was published on 03 April 2023. It is available at https://doi.org/10.1002/cl2.1321.

### 4.1 Criteria for considering studies for this review

#### 4.1.1 Types of studies

The project followed standard procedures for conducting systematic reviews using meta‐analysis techniques.

Randomised and quasi‐randomised controlled trials were eligible. To summarise what is known about the possible causal effects of organised sport participation, we included all study designs that used a control group, that is, a group of children/youth not participating in organised sport. The control group may be offered no treatment or an alternative treatment.

The study designs eligible for inclusion in the review were:

1. Randomised and quasi‐randomised controlled trials (allocated at either the individual level or cluster level, e.g., class/school/geographical area, etc.).
2. Non‐randomised studies (participation has occurred in the course of usual decisions, the allocation to participation in organised sport and no participation is not controlled by the researcher, and there is a comparison of two or more groups of participants, that is, at least a treated group and a control group).

Studies using single group pre‐post comparisons were not eligible for inclusion. Non‐randomised studies using an instrumental variable approach were not eligible for inclusion—see Supporting Information: Appendix 1 (Justification of exclusion of studies using an instrumental variable (IV) approach) for our rationale for excluding studies of these designs. A further requirement to all types of studies (randomised as well as non‐randomised) was that they were able to identify an intervention effect. Studies where, for example, the treatment was offered to children in one school or community only and the comparison group was children at another school/community (or more schools/communities for that matter) cannot separate the treatment effect from the school/community effect.

#### 4.1.2 Types of participants

The review would include young people between 6 and 18 years of age, who either have experienced or are at‐risk of experiencing an adverse outcome such as school failure or drop‐out, substance and/or alcohol abuse, unemployment, long‐term poverty and delinquency/criminal behaviour.

At‐risk may be based on such indicators as the young person's level of association with negative peers (e.g., negative attitudes towards school and poor educational outlook, gang members etc.), hanging out on the streets or in gang neighbourhoods, poor academic history, coming from a highly distressed or crisis ridden, low income family in a racially/ethnically segregated neighbourhood, and prior involvement in illegal and delinquent activities.

Studies where either the majority of participants were between 6 and 18 years of age or studies where a discrete age group within this range was provided were eligible for inclusion.

#### 4.1.3 Types of interventions

The intervention of interest is participation in leisure‐time organised sport. We used the following definition of organised sport: a structured activity through an organisation, requiring physical exertion and/or physical skill and is generally accepted as being a sport. Generally, there is a coach involved, from which participants follow directions and execute the skills taught. The organisation providing the activity may be school or non‐school sports clubs, as long as the activity takes place after school hours. Leisure time physical activity, defined as any unstructured physical activity outside of school hours was not eligible.

Traditional forms of sport provision are youth sport programmes designed to introduce participants to a specific sport that satisfies the desire for belonging, physical fitness, and fun. Quite different from traditional sport programmes are youth sport programmes that make an effort to teach sport skills and life skills, concurrently containing clear expectations for achievement and learning. These programmes are also termed sport‐based youth development interventions (Petitpas et al., 2005). In these programmes, sport is mostly considered a *necessary*, but not *sufficient* condition for the achievement of certain outcomes (Coalter, 2010).

Only the former type of programme was eligible, thus programmes in which sport was augmented with a parallel programme to maximise their potential to achieve certain developmental outcomes were excluded. Also, multiple health behaviour intervention studies (e.g., co‐interventions such as a dietary programme combined with sport) were excluded.

We excluded studies that only addressed ‘exercise’, ‘physical activity’ or ‘physical education’, and not sport. In addition, we excluded studies of yoga and studies of outdoor adventure programmes.

The comparison population was young people at‐risk who did not attend organised sports programmes.

#### 4.1.4 Types of outcome measures

##### Primary outcomes

The primary focus was on measures of problem/high‐risk behaviour, such as delinquency, drug and alcohol use, high levels of externalising problems, school failure, and in the longer run employment, education, training (NEET status). These outcomes had to be measured by self‐reports or reports by authorities, administrative files, registers.

##### Secondary outcomes

A secondary focus was on measures of personal, social and emotional outcomes.

Only valid and reliable outcomes that have been standardised on a different population (and is ‘objective’, i.e., not ‘experimenter‐designed’) was eligible for inclusion. Examples of valid outcomes are measures from the Social Skills Rating System (SSRS; Gresham & Elliot, 1990) or the revision of the SSRS, called the Social Skills Improvement System‐Rating Scales (SSIS‐RS; Gresham & Elliot, 2008), the Strength and Difficulties Questionnaire (SDQ; Goodman, 1997) and the Academic Competence Evaluation Scales (ACES; DiPerna & Elliot, 1999).

Studies were only included if they considered at least one of the primary or secondary outcomes. If it was not clear from the description of outcome measures in the studies whether they were standardised, we used electronic sources to determine whether a measure was standardised or not. We did not consider measures where researchers had picked a subset of questions from a standardised measure.

##### Adverse outcomes

Any adverse effects of interventions were included as an outcome, including a worsening of outcome on any of the included measures.

#### 4.1.5 Duration of follow‐up

Time points planned for measures were:

- While actively engaged in organised sport
- At cessation of participation to 1 year after cessation
- More than 1 year after cessation

There was one controlled trial (non‐randomised) used in the meta analysis, with measures taken at post‐intervention. The remaining studies used in the meta analyses reported the year when the baseline measure of organised sport participation was taken from and the number of years the participants were followed. However, whether the participants continued participating in organised sport throughout the follow‐up period or stopped at some time during the follow‐up period was not reported.

#### 4.1.6 Types of settings

The eligible setting was after‐school sports participation, that is, leisure activities. Public as well as private suppliers, including non‐profit organisations were eligible.

### 4.2 Search methods for identification of studies

The search was performed by two review authors (EB, TF) of which one (EB) is an information specialist.

Relevant studies were identified through electronic searches in bibliographic databases, grey literature repositories and resources, hand search in specific targeted journals, citation tracking, contact to international experts and Internet search engines. A date restriction from 1970 and onwards was applied.

#### 4.2.1 Electronic searches

The following electronic bibliographic databases were searched:

- ERIC (EBSCO) 1970 – March 2023
- Academic Search (EBSCO) 1970 – March 2023
- EconLit (EBSCO) 1970 – March 2023
- PsycINFO (EBSCO) 1970 – March 2023
- SocINDEX (EBSCO) 1970 – March 2023
- International Bibliography of the Social Sciences (ProQuest) 1970 – March 2023
- Sociological Abstracts (ProQuest) 1970 – March 2023
- Science Citation Index Expanded (Web Of Science) 1990 – March 2023
- Social Sciences Citation Index (Web Of Science) 1990 – March 2023
- EMBASE (OVID) 1974 – March 2023

The database searches were performed between 9/3/2023 and 21/3/2023.

##### Description of the search‐string

The search string is based on the PICO(S)‐model, and contains two concepts, of which we have developed two corresponding search facets: population characteristics and the intervention. The search string includes searches in title and abstract as well as subject terms and/or keywords for each facet. The subject terms in the facets were selected according to the thesaurus or index of each database. Keywords were supplied where the search technique provided additional results. Use of wildcards and truncation symbols were used to create searches with multiple spellings or various endings.

##### Example of a search‐string

Below is an exemplified search string utilised to search the database SocINDEX through the EBSCO‐platform. The search string is structured in the following order:

- Search 1–6 covers the intervention
- Search 7–18 covers the population characteristics
- Search 19 combines the two search facets

**EBSCO SocINDEX 1970 − 21 March 2023**. Search modes: Boolean/Phrase. Expanders: Apply equivalent subjects. Limiters: Date of Publication: 19700101 ‐ 20231231.

**Table:** 

#	Searches	Results
S1	TI sport* OR physical act* OR exercise*	13,136
S2	AB sport* OR physical act* OR exercise*	45,380
S3	SU sport* OR physical act* OR exercise*	18,703
S4	S1 OR S2 OR S3	51,526
S5	DE ‘SPORTS ‐‐ Social aspects’ OR DE ‘EXERCISE ‐‐ Social aspects’	429
S6	S4 OR S5	51,526
S7	TI youth OR adolescen* OR young people OR young person* OR young adult* OR teens OR teenager* OR boy* OR girl* OR student*	130,732
S8	AB youth OR adolescen* OR young people OR young person* OR young adult* OR teens OR teenager* OR boy* OR girl* OR student*	312,730
S9	SU youth OR adolescen* OR young people OR young person* OR young adult* OR teens OR teenager* OR boy* OR girl* OR student*	177,403
S10	S7 OR S8 OR S9	354,251
S11	DE ‘YOUTH’ OR DE ‘YOUNG adults’ OR DE ‘YOUNG men’ OR DE ‘YOUNG women’ OR DE ‘ADOLESCENCE’ OR DE ‘TEENAGERS’	43,507
S12	S10 OR S11	355,582
S13	TI vulnerab* OR ‘at risk*’ OR ‘at‐risk*’ OR disaffect* OR ‘youth work*’ OR ‘youth care*’ OR ‘social work*’ OR ‘social care*’ OR underserv* OR depriv* OR minorit* OR ‘low SES’ OR ‘school dropout*’ OR ‘school failure*’ OR disadvantage* OR traumati#* OR maginali#ed OR ‘disruptive behavio#r’ OR abus* OR poverty OR crim* OR homeless* OR street* OR detach* OR delinquen*	204,265
S14	AB vulnerab* OR ‘at risk*’ OR ‘at‐risk*’ OR disaffect* OR ‘youth work*’ OR ‘youth care*’ OR ‘social work*’ OR ‘social care*’ OR underserv* OR depriv* OR minorit* OR ‘low SES’ OR ‘school dropout*’ OR ‘school failure*’ OR disadvantage* OR traumati#* OR maginali#ed OR ‘disruptive behavio#r’ OR abus* OR poverty OR crim* OR homeless* OR street* OR detach* OR delinquen*	480,590
S15	SU vulnerab* OR ‘at risk*’ OR ‘at‐risk*’ OR disaffect* OR ‘youth work*’ OR ‘youth care*’ OR ‘social work*’ OR ‘social care*’ OR underserv* OR depriv* OR minorit* OR ‘low SES’ OR ‘school dropout*’ OR ‘school failure*’ OR disadvantage* OR traumati#* OR maginali#ed OR ‘disruptive behavio#r’ OR abus* OR poverty OR crim* OR homeless* OR street* OR detach* OR delinquen*	320,643
S16	S13 OR S14 OR S15	595,767
S17	DE ‘AT‐risk youth’ OR DE ‘SCHOOL dropouts’ OR DE ‘SCHOOL dropout prevention’ OR DE ‘YOUNG people not in education, employment, or training’ OR DE ‘SOCIOECONOMICALLY disadvantaged students’ OR DE ‘POOR youth’ OR DE ‘STREET youth’ OR DE ‘HOMELESS youth’ OR DE ‘DELINQUENT youths’	2237
S18	S16 OR S17	595,825
S19	S6 AND S12 AND S18	2968
S20	S6 AND S12 AND S18 – *Limiters – Date of Publication: 19700101‐20231231*	2864

#### 4.2.2 Searching other resources

##### Hand‐search

We conducted a hand search of specific journals, to make sure that all relevant articles were found. The hand search focused on editions published between 2018 and 2023 to secure recently published articles which have not yet been indexed in the bibliographic databases (note that in the protocol (Filges, 2023) unfortunately, it says ‘secure recently unpublished’ wich is a mistake, it should be ‘published’).

Four specific journals, based on the identified records from the electronic searches, were hand‐searched in the time period between 2018 and March 2023:

- Children and Youth Services
- The Future of Children
- Research on Social Work Practice
- Journal of Prevention and Intervention in the Community

##### Searches for unpublished literature in general

The searches on other resources and for unpublished literature were done between 03/05/2023 and 23/05/2023. Most of the resources searched for unpublished literature include multiple types of references. As an example, the resources listed to identify reports from national bibliographical resources also include working papers and dissertations, as well as peer‐reviewed references and documents from preprint databases. In general, there is a great amount of overlap between the types of references in the chosen resources. The resources are listed once under the category of literature we expect to be most prevalent in the resource, even though multiple types of unpublished/published literature might be identified in the resource. Terms used to search other resources were based on the general search strategy. Combinations of terms such as ‘sport’ with terms for the population (i.e., youth or at‐risk) were utilised. All of these searches can be seen in Supporting Information: Appendix 2.

##### Search for dissertations

We searched the following resources for dissertations:

- EBSCO Open Dissertations (EBSCO‐host) – https://biblioboard.com/opendissertations/
- OATD – Open Access Theses and Dissertations – oatd.org

##### Search for working papers/conference proceedings

We searched the following resources for working papers/conference proceedings:

- American Institutes for Research (AIR) – https://www.air.org/
- *Manpower Demonstration Research Corporation* (MDRC) – https://www.mdrc.org/
- Urban Institute – https://www.urban.org/
- Google Scholar – https://scholar.google.com
- Google – https://google.com

##### Search for preprints/post prints/working papers

We searched the following resources for preprints, postprints, working papers and published papers:

- SportRxiv – Open access subject repository of preprints, post prints within sport, exercise, performance, and health research – https://sportrxiv.org/
- SocArxiv – Open archive of the social sciences of working papers, preprints, and published papers – https://osf.io/preprints/socarxiv
- PsyArXiv – Open archive of preprints, working manuscripts, and post prints in the psychological sciences – https://psyarxiv.com
- OSF Preprints – Open source multidisciplinary preprints as well as post prints and working papers – https://osf.io/preprints/

##### Search for reports

We searched for Public/Private Ventures (P/PV) reports (although Public/Private Ventures (P/PV) has ceased operations, its publications are archived with the Foundation Center's IssueLab: https://ppv.issuelab.org.

##### Search for non‐US literature

We searched the following resources for non‐US literature:

- Research Portal Denmark – https://forskningsportal.dk/
- SwePub – Academic publications at Swedish universities – http://swepub.kb.se/
- NORA – Norwegian Open Research Archives – http://nora.openaccess.no/
- CORE – research outputs from international repositories – https://core.ac.uk/
- Skolporten – Swedish Dissertations – https://www.skolporten.se/forskning/
- DIVA – Digital Scientific Archives – http://www.diva-portal.org/smash/

##### Search for systematic reviews

Relevant systematic reviews identified during the search process were used for citation‐tracking, to extract relevant references from the review.

##### Citation‐tracking

To identify both published studies and grey literature, we utilised citation‐tracking/snowballing strategies. Our primary strategy was to citation‐track related systematic‐reviews and meta‐analyses. The review team also checked reference lists of included primary studies for new leads.

##### Contact to experts

By e‐mail during May 2023, we contacted international experts to identify unpublished and ongoing studies.

##### Other criteria

Studies were not excluded based on publication status or language (although the ability to assess the relevance of studies was limited by the language skills in the review team). Studies authored before 1970 were not eligible.

### 4.3 Data collection and analysis

#### 4.3.1 Selection of studies

In pairs of two, one review author and two review team assistants first independently screened titles and abstracts to exclude studies that were clearly irrelevant. Studies considered eligible or studies where there was insufficient information in the title and abstract to judge eligibility, were retrieved in full text. The full texts were then screened independently by one review author and one review team assistant. Any disagreement about eligibility was resolved by discussion. Exclusion reasons for studies that otherwise might be expected to be eligible were documented and presented in section *Excluded studies*.

**Figure 1:**
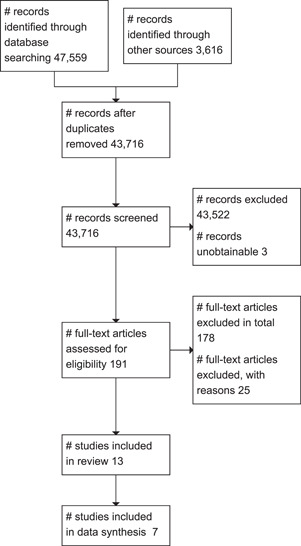
Flow diagram.

The study inclusion criteria were piloted by the review author and the team assistant (see Supporting Information: Appendix 3). The overall search and screening process is illustrated in Figure 1. None of the review authors or team members were blind to the authors, institutions, or the journals responsible for the publication of the articles.

#### 4.3.2 Data extraction and management

Two review authors independently coded and extracted data from the included studies. A coding sheet was piloted and revised as necessary (see Supporting Information: Appendix 4). Disagreements were minor and resolved by discussion. Data and information were extracted on: available characteristics of participants, intervention characteristics and control conditions, research design, sample size, risk of bias and potential confounding factors, outcomes, and results. Analysis was conducted in RevMan. Extracted numerical and descriptive data, and the risk of bias assessments described in the next section, can be found in the supplementary documents.

#### 4.3.3 Assessment of risk of bias in included studies

We assessed the risk of bias in randomised studies using Cochrane's revised risk of bias tool, ROB 2 (Higgins et al., 2019).

The tool is structured into five domains, each with a set of signalling questions to be answered for a specific outcome. The five domains cover all types of bias that can affect results of randomised trials.

The five domains for individually randomised trials are:

(1) bias arising from the randomisation process;
(2) bias due to deviations from intended interventions (separate signalling questions for effect of assignment and adhering to intervention);
(3) bias due to missing outcome data;
(4) bias in measurement of the outcome;
(5) bias in selection of the reported result.
  For cluster‐randomised trials, an additional domain was included ([1b] Bias arising from identification or recruitment of individual participants within clusters). We used the latest template for completion (currently it is the version of 15 March 2019 for individually randomised parallel‐group trials and 20 October 2016 for cluster randomised parallel‐group trials). In the cluster randomised template, however, only the risk of bias due to deviation from the intended intervention (effect of assignment to intervention; intention to treat ITT) is present and the signalling question concerning the appropriateness of the analysis used to estimate the effect is missing. Therefore, for cluster randomised trials we only used the signalling questions concerning the bias arising from identification or recruitment of individual participants within clusters from the template for cluster randomised parallel‐group trials; otherwise we used the template and signalling questions for individually randomised parallel‐group trials.
  We assessed the risk of bias in non‐randomised studies, using the model ROBINS–I, developed by members of the Cochrane Bias Methods Group and the Cochrane Non‐Randomised Studies Methods Group (Sterne et al., 2016a). We used the latest template for completion (currently it is the version of 19 September 2016).
  The ROBINS‐I tool is based on the Cochrane RoB tool for randomised trials, which was launched in 2008 and modified in 2011 (Higgins et al., 2011).
  The ROBINS‐I tool covers seven domains (each with a set of signalling questions to be answered for a specific outcome) through which bias might be introduced into non‐randomised studies:
  (1) bias due to confounding
  (2) bias in selection of participants
  (3) bias in classification of interventions
  (4) bias due to deviations from intended interventions;
  (5) bias due to missing outcome data;
  (6) bias in measurement of the outcome;
  (7) bias in selection of the reported result.

The first two domains address issues before the start of the interventions and the third domain addresses classification of the interventions themselves. The last four domains address issues after the start of interventions and there is substantial overlap between these four domains between bias in randomised studies and bias in non‐randomised studies trials (although signalling questions are somewhat different in several places, see Higgins et al., 2019; Sterne et al., 2016b).

Randomised study outcomes are rated on a ‘Low/Some concerns/High’ scale on each domain, whereas non‐randomised study outcomes are rated on a ‘Low/Moderate/Serious/Critical/No Information’ scale on each domain. The level ‘Critical’ means: the study (outcome) is too problematic in this domain to provide any useful evidence on the effects of intervention, and it is excluded from the data synthesis. The same critical level of risk of bias (excluding the result from the data synthesis) is not directly present in the RoB 2 tool, according to the guidance to the tool (Higgins et al., 2019).

In the case of an RCT, where there is evidence that the randomisation has gone wrong or is no longer valid, we assessed the risk of bias of the outcome measures using ROBINS‐I instead of ROB 2. Examples of reasons for assessing RCTs using the ROBINS‐I tool may include studies showing large and systematic differences between treatment conditions while not explaining the randomisation procedure adequately, suggesting that there was a problem with the randomisation process; studies with large scale differential attrition between conditions in the sample used to estimate the effects; or studies selectively reporting results for some part of the sample or for only some of the measured outcomes. In such cases, differences between the treatment and control conditions are likely systematically related to other factors than the intervention and the random assignment is, on its own, unlikely to produce unbiased estimates of the intervention effects. Therefore, as ROBINS‐I allow for an assessment of, for example, confounding, we believe it is more appropriate to assess effect sizes from studies with a compromised randomisation using ROBINS‐I than ROB 2. Like other effect sizes assessed with ROBINS‐I, these effect sizes may receive a ‘Critical’ rating and thus be excluded from the data synthesis. None of the studies were moved from ROB 2 to ROBINS‐I.

We stopped the assessment of a non‐randomised study outcome as soon as one domain in the ROBINS‐I was judged as ‘Critical’.

‘Serious’ risk of bias in multiple domains in the ROBINS‐I assessment tool may lead to a decision of an overall judgement of ‘Critical’ risk of bias for that outcome, and it will be excluded from the data synthesis.

##### Confounding

An important part of the risk of bias assessment of non‐randomised studies is consideration of how the studies deal with confounding factors. Systematic baseline differences between groups can compromise comparability between groups. Baseline differences can be observable (e.g., age and gender) and unobservable (to the researcher; e.g., motivation and ‘ability’). There is no single non‐randomised study design that always solves the selection problem. Different designs represent different approaches to dealing with selection problems under different assumptions, and consequently require different types of data. There can be particularly great variations in how different designs deal with selection on unobservables. The ‘adequate’ method depends on the model generating participation, that is, assumptions about the nature of the process by which participants are selected into a programme.

A major difficulty in estimating causal effects of sports participation is the potential endogeneity of the young individual's life circumstance and developmental state that leads to the decision to participate in organised sport and, if not accounted for, it will yield biassed estimates.

As there is no universal correct way to construct counterfactuals for non‐randomised designs, we looked for evidence that identification was achieved, and that the authors of the primary studies justified their choice of method in a convincing manner by discussing the assumption(s) leading to identification (the assumption(s) that make it possible to identify the counterfactual). Preferably, the authors should make an effort to justify their choice of method and convince the reader that the only difference between a treated individual and a non‐treated individual is the treatment. The judgement is reflected in the assessment of the confounder unobservables in the list of confounders considered important at the outset (see Supporting Information: Appendix 5).

In addition to unobservables, we had identified the following observable confounding factors to be most relevant: age, gender and risk indicators as described in section *Type of participants*. In each study, we assessed whether these indicators had been considered, and in addition, we assessed other factors likely to be a source of confounding within the individual included studies.

##### Importance of pre‐specified confounding factors

The motivation for focusing on age, gender and risk indicators is given below.

The prevalence of different types of behavioural and psychological problems, coping skills, cognitive and emotional ability vary throughout a child's development through puberty and into adulthood (Cole et al., 2005), and therefore we consider age to be a potential confounding factor. Furthermore, there are substantial gender differences in behaviour problems, coping and risk of different types of adverse outcomes, which is why we also include gender as a potential confounding factor (Card et al., 2008; Hampel & Petermann, 2005; Hart et al., 2007).

Pre‐treatment group equivalence of risk indicators is indisputable an important confounder as young people in stable life circumstances, typically are not at risk of developing the range of problems we will consider in this review. Therefore, the accuracy of the estimated effects of sports programmes will depend crucially on how well the risk indicators are controlled for.

##### Effect of primary interest and important co‐interventions

We were mainly interested in the effect of starting and adhering to the intended intervention, that is, the treatment on the treated (TOT) effect. The risk of bias assessments was therefore performed in relation to this specific effect.

The risk of bias assessments of both randomised trials and non‐randomised studies considered adherence and differences in additional interventions (‘co‐interventions’) between intervention groups. Relevant co‐interventions are those that individuals might receive with or after starting the intervention of interest and that are both related to the intervention received and prognostic for the outcome of interest. Important co‐interventions we considered were interventions delivered as part of sport‐based youth development programmes. These programmes may be explicitly teaching personal and social responsibility or other life skills. Although these types of programmes were not eligible (see section *Types of interventions*), we carefully considered if there were any co‐interventions teaching other than the sport discipline in question.

##### Assessment

Two review authors independently assessed the risk of bias for each relevant outcome from the included studies. We discussed all initial disagreements and were able to reach a consensus in all cases. We report the risk of bias assessment in risk of bias tables for each included study outcome in a supplementary document.

#### 4.3.4 Measures of treatment effect

Reported effect sizes that could not be pooled (were reported in a single study only) were reported in as much detail as possible. Software for storing data and statistical analyses were RevMan 5.0 and Excel.

##### Continuous outcomes

We calculated effects sizes with 95% confidence intervals (CIs), where means and standard deviations were available, or alternatively from mean differences and standard deviations of the mean or reported effect sizes and 95% CIs (whichever were available), using the methods suggested by Lipsey and Wilson (2001).

##### Dichotomous outcomes

For the dichotomous outcomes (depression diagnosis and symptoms, anxiety diagnosis and substance use), we used odds ratios with 95% confidence intervals.

#### 4.3.5 Unit of analysis issues

We checked for consistency in the unit of allocation and the unit of analysis, as statistical analysis errors can occur when they are different. There were no studies where the unit of allocation differed from the unit of analysis. In one study, however, the treatment was delivered in a group setting, and the study investigators had not applied appropriate analysis methods that control for clustering effects (see Pals et al., 2008).

We adjusted the effect size and its standard error using the methods suggested by Hedges (2007), using an ICC of 0.02 (we searched the literature for estimates of relevant ICC's; Campbell, 2000; Connolly, 2018; Health Services Research Unit, 2004; Parker, 2021; Stallard, 2012), and assumed equal cluster sizes. To calculate an average cluster size, we divided the total sample size in the study by the number of clusters.

In the sensitivity analysis, we report both results using unadjusted effect sizes and using a substantially higher ICC (0.3) than in the primary analysis.

#### 4.3.6 Criteria for determination of independent findings

To determine the independence of results in included studies, we considered whether individuals may have undergone multiple interventions, whether there were multiple treatment groups, whether several studies are based on the same data source and whether studies report multiple conceptually similar outcomes.

##### Multiple intervention groups and multiple interventions per individuals

One study had multiple intervention groups with different individuals as results were reported separated by ethnicity. As there were not enough studies to apply robust standard errors (Hedges et al., 2010), we used a synthetic effect size (the average) to avoid dependence between effect sizes.

##### Multiple studies using the same sample of data

Two studies (Easterlin, 2019; Hull, 2008) used the same data set (individuals who participated in wave 1 (1994–1995) of the National Longitudinal Study of Adolescent to Adult Health). The results of both studies were used in separate parts of the data synthesis as one of these studies reported 1 year follow‐up outcomes and the other reported 13 year follow‐up outcomes.

##### Multiple time points

When the results were measured at multiple time points, each outcome at each time point was analysed in a separate meta‐analysis with other comparable studies taking measurements at a similar time point. The measures were taken at different time points, either reported at postintervention, or between 1 and 13 years follow up. Due to the small number of studies available for meta‐analysis, we grouped the outcomes at post intervention and follow up.

##### Multiple conceptually similar outcomes

None of the studies used in meta analyses reported multiple estimates of effects regarding the same/similar outcome.

#### 4.3.7 Dealing with missing data

None of the studies used in the data synthesis had missing summary data.

#### 4.3.8 Assessment of heterogeneity

Heterogeneity amongst primary outcome studies was assessed with the Chi‐squared (Q) test, and the I‐squared, and τ‐squared statistics (Higgins et al., 2003). Any interpretation of the Chi‐squared test was made cautiously on account of its low statistical power.

#### 4.3.9 Assessment of reporting biases

Reporting bias refers to both publication bias and selective reporting of outcome data and results. Here, we state how we planned to assess publication bias. We planned to use funnel plots for information about possible publication bias. However, we did not find sufficient studies (Higgins & Green, 2011).

#### 4.3.10 Data synthesis

Meta‐analysis of outcomes was conducted on each metric (as outlined in section ‘*Types of outcome measures*’) separately, and we performed separate analyses for short‐term and long‐term outcomes.

Studies that were coded with a Critical risk of bias were not included in the data synthesis.

As the intervention dealt with diverse populations of participants (from different countries, facing different life circumstances, etc.), and we therefore expected heterogeneity amongst primary study outcomes, all analyses of the overall effect were inverse variance weighted using random effects statistical models that incorporate both the sampling variance and between study variance components into the study level weights. The estimation of *τ* ^2^ was the DerSimonian and Laird (1986) estimate. Random effects weighted mean effect sizes were calculated using 95% CIs, and we provide graphical displays (forest plots) of effect sizes.

None of the studies that could be used in a particular meta‐analysis used the same data. One study provided results separated by subscales of an outcome measurement. As there was not a sufficient number of studies included in any of the meta‐analyses to use robust variance estimation as planned (Filges, 2023), we conducted the meta‐analyses using a synthetic effect size (the average of the subscales for a particular measurement) to avoid dependence between effect sizes.

All meta‐analyses were carried out in Revman 5.4.

#### 4.3.11 Subgroup analysis and investigation of heterogeneity

There were not enough studies to perform moderator analyses.

#### 4.3.12 Sensitivity analysis

Sensitivity analysis was carried out where possible, by restricting the meta‐analysis to a subset of all studies included in the original meta‐analysis. We considered sensitivity analysis for each domain of the risk of bias checklists and restricted the analysis to studies with a low risk of bias. Sensitivity analysis was only conducted when there were more than two studies left in the analyses.

We tested sensitivity to clustered delivery of treatment by reporting results using both unadjusted effect sizes and using a substantially higher ICC (0.3) than in the primary analysis.

#### 4.3.13 Treatment of qualitative research

We did not include qualitative research.

#### 4.3.14 Summary of findings and assessment of the certainty of the evidence

We did not plan to include Summary of findings and assessment of the certainty of the evidence.

## 5 RESULTS

### 5.1 Description of studies

#### 5.1.1 Results of the search

We summarise the search results in a flow chart in Figure 1. The total number of potential relevant studies was 43,716 after excluding duplicates (database: 40,100, grey, snowballing and other resources: 3616). We screened all studies based on title and abstract; 43,522 were excluded for not fulfilling the screening criteria, three studies were unobtainable despite efforts to locate them through libraries and searches on the Internet (they are listed in Table 1) and 191 studies were ordered, retrieved, and screened in full text. Of these, 178 did not fulfil the screening criteria and were excluded. We included a total of 13 studies in the review. The references are listed in section *References to included studies*.

**Table 1:** Unobtainable studies.

Study
Nam H., Kang K. and Lee A. Effects of an Afterschool Sports Program on At‐Risk Youth in Korea. *Research Quarterly for Exercise and Sport*. 2014, 85, pp. 81–82.
Noel‐London Kemba C. Tackling health equity through sports and sports medicine: The intersection of public health policy and allied health. *Dissertation Abstracts International: Section B: The Sciences and Engineering*. 2022.
Segrave Jeffrey O. Athletic Delinquency: A Preliminary Approach. Paper presented at the Annual Convention of the American Alliance for Health, Physical Education, and Recreation (94th, New Orleans, Louisiana, March 17, 1979).

#### 5.1.2 Included studies

The search and screening resulted in a final selection of 13 studies which met the inclusion criteria for this review. All 13 studies were non‐randomised studies. The studies were carried out in five different countries (Australia, Canada, Peru, Sweden and US) with the majority from the US. Descriptions of the intervention and control conditions within each included study were extracted in as much detail as possible and can be found in the supplementary descriptive table.

In Table 2 we show the total number of studies that met the inclusion criteria for this review. The first column shows the total number of studies grouped by country of origin. The second column shows the number of these studies that did not provide data to calculate an effect estimate. The third column gives the number of studies that were coded with Critical risk of bias. The last column gives the total number of studies used in the data synthesis.

**Table 2:** Number of included studies by country.

Country	Total	Reduction due to		
		Missing data	Critical risk of bias	Used in data synthesis
Canada	2			2
USA	8	1	3	4
Peru	1		1	
Sweden	1	1	1	
Australia	1			1
Total	13	2	5	7

*Note*: One study was both rated Critical risk of bias and had missing data.

Five studies could not be used in the data synthesis as all reported outcomes were judged to have a critical risk of bias. (see supplementary documents for the detailed risk of bias assessments). In accordance with the protocol, we excluded studies rated overall Critical risk of bias items from the data synthesis on the basis that they would be more likely to mislead than inform.

One study (Pitt, 2015) did not report data in a form that enabled the calculation of effect sizes and standard errors (reported results from a Tobit Regression with two types of treatments, contact sport and non‐contact sport).

All studies are listed in Table 3 along with the reason why the study was not used in the data synthesis.

**Table 3:** Characteristics of included studies.

Study	Outcome	Country	Used in data synthesis/reason not used
Afifi (2022)	Substance use (prevalence of past 30‐day substance use: cigarette, alcohol, and cannabis)	Canada	Used in data synthesis
Antonio (2016)	Criminal record	Peru	Rated Critical risk of bias
Brière (2018)	Depressive symptoms, social anxiety symptoms, loneliness	Canada	Used in data synthesis
Chang (2021)	Suspension and dropout	US	Rated Critical risk of bias
D'Andrea (2013)	Child Behavior Check List (CBCL total and internalising/externalising subscales)	US	Used in data synthesis
Easterlin (2019)	Having ever received a diagnosis of depression, having ever received a diagnosis of anxiety, and screening positive for current depressive symptoms	US	Used in data synthesis
Hastad (1984)	Deviant behaviour (drug related, school related, non‐school related and a composite)	US	Rated Critical risk of bias
Hull (2008)	Emotional distress, positive well‐being	US	Used in data synthesis
Kwak (2017)	Delinquency, depressive symptoms, trauma symptoms	US	Used in data synthesis
Lundkvist (2020)	Antisocial behaviours (e.g., aggressiveness, conflicts with peers, family and friends, concentration problems and rowdy behaviours like deliberately damaging others possessions or carrying a knife)	Sweden	Rated Critical risk of bias and cannot calculate effect size and SE
McHale (2005)	Self‐esteem	US	Rated Critical risk of bias
O'Donnell (2020)	Emotional and behavioural difficulties from the Strengths and Difficulties Questionnaire (SDQ); A total difficulties score was computed from 20 items designed to measure emotional symptoms, conduct problems, hyperactivity, and peer relationship problems	Australia	Used in data synthesis
Pitt (2015)	Marijuana use, alcohol use, non‐violent delinquency, and violent delinquency	US	Cannot calculate effect size and SE

Of the seven studies available for data synthesis, however, one study did not have any outcomes in common with the other six studies and one study did not report the outcome at a time point in common with the other six studies. The effect sizes from these two studies could thus not be used in any of the meta‐analyses. The outcomes are reported in Table 7 along with other outcomes from the other five studies that could not be pooled as they also were only reported in a single study.

The main characteristics of the seven studies used in the data synthesis are shown in Table 4.

**Table 4:** Characteristics of studies used in data synthesis.

Characteristic (Number of studies reporting)		
Year start of participation (*N* = 6)	Average (SD)	2007 (9.93)
	Range	1995–2018
Number of participants, treated (*N* = 7)	Average (SD)	2450.7 (4376.08)
	Range	28–12,110
Number of participants, control (*N* = 7)	Average (SD)	1380.57 (1963.32)
	Range	26–5440
Number of participants, total (*N* = 7)	Average (SD)	3831.29 (6312.69)
	Range	54–17,550
Percent female (*N* = 6)	Average (SD)	59.89 (18.06)
	Range	47.7–100
Mean age (*N* = 5)	Average (SD)	14.61 (0.85)
	Range	13.30–15.52
Percent white (*N* = 5)	Average (SD)	44.62 (32.12)
	Range	0–85
Country (*N* = 7)	Australia	1 (14%)
	Canada	2 (29%)
	USA	4 (57%)
Risk indicator (*N* = 7)	History of or exposure to Adverse Childhood Experiences (ACEs)	4 (57%)
	Living in disadvantaged neighbourhoods	1 (14%)
	Being refugee	1 (14%)
	Ethnicity (Black or Hispanic)	1 (14%)
Sport characteristic (*N* = 7)	Team	2 (29%)
	Individual	1 (14%)
	Varies	2 (29%)
	Not reported	3 (43%)
Type of programme (*N* = 7)	Intervention	1 (14%)
	Participation in general	6 (86%)

*Note*: The type of sport sums to more than 100% as one study reported both team and individual sport participation.

The timespan of the start year of the interventions analysed in included studies is 23 years, from 1995 to 2018 and, on average, the intervention start year was 2007 and the median start year was 2008. Two studies were carried out in Canada, one in Australia, and the remaining in the USA. The average number of participants in sports analysed was 2451, ranging from 28 to 12,110 per study with a median of 316. The average number of controls was 1381, ranging from 26 to 5440 per study with a median of 452. The average age of sport participants was 14.6 years ranging from 13.3 to 15.5 years. On average, females constituted more than half of sports participants, 60%, ranging from 48% to 100%. Ethnicity of sport participants was reported in only five studies and the average percent of white was 45% with great variation, ranging from 0% to 85%. The risk indicators of the population analysed were a history of or exposure to Adverse Childhood Experiences (ACEs) in four studies, living in disadvantaged neighbourhoods in one study, being a refugee in one study, and ethnicity (Black or Hispanic) in one study. One study reported that the type of sport was competitive team with limited contact, another study reported that 75% were team and 25% individual sport, and the remaining studies either reported that the type of sport varied or did not report what type of sport was analysed. A single study analysed a sport intervention, named Do the Good (DtG). Participants (all girls who resided in residential treatment facilities), attended a once‐weekly hour‐long basketball game played against a competing residential treatment facility team over a 5‐month season (D'Andrea, 2013).

#### 5.1.3 Excluded studies

In addition to the 13 studies that met the inclusion criteria for this review, 25 studies at first sight appeared relevant but did not meet our criteria for inclusion. The studies and reasons for exclusion are given in Table 5. More than half (15 studies) were excluded because either the intervention and outcome were measured at the same time or outcome was measured before intervention.

**Table 5:** Studies excluded with reason.

Study	Reason for exclusion
Blomfield (2010)	Treatment and outcomes are measured in the same time period
Bonhauser et al. (2005)	Not an after‐school intervention
Burton (2005)	Treatment and outcomes are measured in the same time period
Crean (2012)	Treatment and outcomes are measured in the same time period
Dawkins (2006)	Treatment and outcomes are measured in the same time period
Felfe (2011)	Treatment and outcomes are measured in the same time period
Hallingberg (2015)	Treatment and outcomes are measured in the same time period
Jiang (2012)	Treatment and outcomes are measured in the same time period
Kauh (2011)	The intervention is AfterZone, three types of activities (sports, skills and arts) and they are not reported separately and the conrol group participates in sports outside of the intervention
Lester (2017)	Outcome is measured before the intervention
Long (2004) Table 40 p. 184 has only blacks	Treatment and outcomes are measured in the same time period
Nicholson (2019)	Analyses a variety of sports diciplines and the comparison is all that do not participate in that particular sport discipline
Matta (2021)	Treatment and outcomes are measured at the same time
Melnick (1992)	Participation groups: participation in both sophomore and senior year, participation in sophomore but not senior year, non‐participant in sophomore and participant in senior year, senior nonvarsity nonleader and sophomore participant, senior nonvarsity leader and sophomore participant, senior nonvarsity leader and sophomore participant, senior varsity nonleader and sophomore participant, and senior varsity leader and sophomore participant. Compares the first group (participation in both sophomore and senior year) to all others merged
Metzger (2009)	Effect of sport is not isolated (cluster analysis where sport participation is included in several of the profiles)
Montoya (2012)	The sample is divided into four groups: sport participation only, other activities only, multiple activities and no participation, thus cannot identify the effect of sport participation vs. non‐participant
Palermo et al. (2006)	No relevant outcomes (only The Carey Temperament Scale [Carey Temperament Scales, B‐DI 14636N. 55th St., Scottsdale, AZ 85254]) was used to determine the child's temperamental style on the domains of intensity (defined as the energy level of behaviour responses, regardless of quality or direction); adaptability (or the ease or difficulty with which reactions to stimuli can be modified in a desired way); and mood (i.e., the amount of pleasant or unpleasant behaviour observed in various situations)
Ryan (2017)	Treatment and outcomes are measured at the same time
Sabo (1986)	Compares athletes defined as those who participated on varsity teams both in their sophomore and their senior year to non‐athletes defined as those who participated only in sophomore year or not at all
Super (2018)	Sport participation and outcome measured at both T1 and T2, analyse the relation between participation at T1 on T1 outcome and participation at T2 on T2 oucome only, thus treatment and outcomes are measured in the same time period
Taylor (2010)	Do not report whether sport participation is based on a retrospective question or if it is current participation, but most likely it is current. Relevant outcomes are lifetime (delinquency) or last 30 days (alcohol use)
Taylor (2012)	Not sport vs. not sport but: In order, responses were placed along a continuum of participation: none (0), informal only (low, 1), school only (medium, 2), and school and informal (high, 3) and most likely analyses a level variable (it is a bit unclear)
Taylor (2016)	Do not report whether sport participation is based on a retrospective question or if it is current participation, but most likely it is current. Relevant outcomes are lifetime (delinquency) or last 30 days (alcohol use)
Terry et al. (2014)	School‐based intervention delivered during lunchtime
Yin (1999)	Sport participation is current and outcome (delinquency acts) is during past 12 months

It is not possible to identify a causal effect when all variables used (treatment, confounders and outcome) are measured at the same time or when outcome measures predates the intervention. Standard accounts of causality assume that there is a temporal order (Intervention predates outcome) of the variables in the analysis. Lacking temporal information, it is impossible to decide which of two dependent variables is the cause and which the effect (Pearl, 2009).

Other reasons were no relevant outcome (one study), the intervention analysed was not participation in organised sport as defined in this review (two studies), and the effect of sport participation was not analysed or reported separately (seven studies).

### 5.2 Risk of bias in included studies

The risk of bias coding for each of the 13 studies and their outcomes is shown in a supplementary document.

All studies used non‐randomised designs, and were rated using the ROBINS‐I tool. Table 6 shows a summary of the risk of bias associated with the studies. As stated in the protocol, we stopped the assessment of a non‐randomised study outcome when it was rated ‘Critical’ on any of the items. Therefore, not all studies are rated on all domains.

**Table 6:** Summary risk of bias, non‐randomised studies.

	Low risk of bias	Moderate risk of bias	Serious risk of bias	Critical risk of bias	No information	Not rated
Overall judgement	0	2	6	5	0	0
Confounding bias	1	1	6	5	0	0
Selection bias	6	2	0	0	0	5
Classification bias	2	6	0	0	0	5
Deviation bias	1	2	0	0	5	5
Missing data	2	3	0	0	3	5
Measurement of Outcome	6	2	0	0	0	5
Selection of Reported Results	0	8	0	0	0	5

Five studies were rated Critical risk of bias on the Overall judgement item, corresponding to a risk of bias so high that the findings should not be considered in the data synthesis. The overall Critical risk of bias rating was due to issues on the Confounding bias item; all five were rated Critical risk of bias on this item; that is, they failed to establish a comparison group that was balanced on important confounders and further either did not control for any confounders or included bad controls.

Six studies were rated Serious risk of bias overall and two studies were rated Moderate risk of bias overall. Unfortunately, one of the studies rated Moderate risk of bias overall (Pitt, 2015) did not report data that permitted calculation of an effect size and standard error. A Tobit regression model was applied, and the coefficients reported can not be interpreted as the effect of sports participation on the outcomes. Instead, they should be interpreted as the combination of (1) the change in outcome of those above the chosen limit, weighted by the probability of being above the limit; and (2) the change in the probability of being above the limit, weighted by the expected value of the outcome if above (McDonald, 1980). This left only seven studies to be used in the data synthesis.

Of the eight studies not rated Critical risk of bias overall, six studies had serious issues on the Confounding item, one had Moderate issues and one was rated Low risk of bias. On the Selection bias item, six were rated Low risk of bias and two were rated Moderate risk of bias. Two studies were rated Low risk of bias on the Classification item and six were rated Moderate risk of bias; one was rated Low risk of bias on the Deviation item, two were rated Moderate and five studies did not provide enough information for us to rate on this item. Likewise, three studies did not provide enough information to be rated on the Missing data item, whereas two were rated Low risk of bias and three were rated Moderate risk of bias. On the Measurement item, six were rated Low risk of bias and two were rated Moderate risk of bias. All eight studies were rated Moderate risk of bias on the Selection of Reported Results mainly because there was no a priori analysis plan.

#### 5.2.1 Synthesis of results

Seven studies were not rated Critical risk of bias and reported data that permitted calculation of an effects size and standard error and could thus be used in the data synthesis.

A large variety of different outcomes were reported in the studies (e.g., substance use, delinquency, mental health and psychosocial adjustment).

To carry out a meta‐analysis, every study must have a comparable effect size. We synthesise effects separately by type of outcome (conceptual outcomes as outlined in section ‘*Types of outcomes measures*’) and time point (end of intervention and follow up). Unfortunately, each type of outcome was only reported in a small subset of studies (in many cases, in only a single study). It was therefore only possible to pool effect sizes in two meta‐analyses and, further, each meta analysis contains a very small number of effect sizes, two respectively three effect sizes. The studies included in the meta‐analyses contribute only a single effect size to each analysis.

All continuous outcomes (effect sizes measured as Hedges *g*) were coded such that a larger effect size indicated better outcomes for the treated group. All binary outcomes (reported as odds ratio) were likewise coded such that a larger effect size indicated better outcomes for the treated group.

##### Primary outcomes

A number of outcomes were reported in a single study only. The outcomes were measures on substance use and delinquency/criminal behaviour. The effect sizes and 95% CIs are reported in Table 7.

**Table 7:** Additional primary and secondary outcomes.

	Overall psychosocial adjustment			Hedges *g*	SE
Study	Measure	Outcome	Time	Effect size	95% CI
D'Andrea (2013)	CBCL	Internalising	Post	1.82	[1.19, 2.45]
D'Andrea (2013)	CBCL	Externalising	Post	−0.14	[−0.67, 0.38]
Brière (2018)	SCAS	Social anxiety	Post	0.04	[0.01, 0.07]
Brière (2018)	MASPAQ	Loneliness	Post	0.09	[0.05, 0.12]

	Delinquency			Hedges *g*	
Kwak (2017)	Modified Self Report of Delinquency scale	Delinquency	0–3 years FU	0.01	[−0.16, 0.17]

	Depression/anxiety			OR	95% CI
Easterlin (2019)	Self‐reported	Depression diagnosis	0–13 years FU	1.25	[0.98, 1.59]
Easterlin (2019)	Self‐reported	Anxiety diagnosis	0–13 years FU	1.41	[1.10, 1.81]
Easterlin (2019)	CES‐D–10	Current depressive symptoms	13 years FU	1.17	[0.98, 1.40]

	Substance use			OR	95% CI
Afifi (2022)	Self‐reported	Cigarette/alcohol/cannabis use past 30 days. Less than once a week	0–1 year FU	0.80	[0.40, 1.59]
Afifi (2022)	Self‐reported	Cigarette/alcohol/cannabis use past 30 days. 1 to 3 times per week	0–1 year FU	1.06	[0.69, 1.64]
Afifi (2022)	Self‐reported	Cigarette/alcohol/cannabis use past 30 days. 4 or more times per week	0–1 year FU	1.39	[0.87, 2.22]

*Note*: Positive continuous effect size (Hedges *g*) favours sport participants, OR greater than one favours sport participants.

##### Secondary outcomes

Two studies analysed the effect of organised sport participation on overall psychosocial adjustment at post‐intervention. Measures used were: The Strengths and Difficulties (SDQ) and Child Behavior Check List (CBCL).

**Figure 2:**
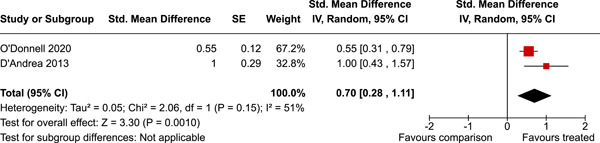
Forest plot Overall psychosocial adjustment.

The random effects weighted standardised mean difference at post intervention was 0.70 (95% CI 0.28–1.11) and statistically significant. The forest plot is displayed in Figure 2. There was a small amount of heterogeneity between the two studies; the estimated *τ* ^2^ was 0.05, *Q* = 2.06, df = 1 and *I* ^2^ was 51% as displayed in Figure 2. Prediction intervals were not calculated with only two studies in the analysis (Higgins, 2009).

Three studies analysed the effect of organised sports participation on depressive symptoms at 0–3 years follow‐up. Measures used were Center for Epidemiological Studies‐Depression (CES‐D) and Children's Depression Inventory (CDI).

**Figure 3:**
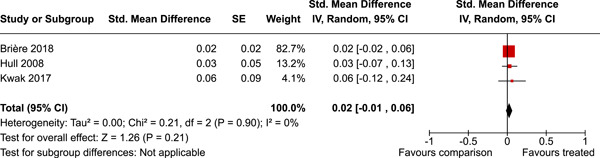
Forest plot Depressive symptoms.

The random effects weighted standardised mean difference at 0–3 years follow‐up was 0.02 (95% CI −0.01 to 0.06) and not statistically significant. The forest plot is displayed in Figure 3. There was no heterogeneity between the three studies; the estimated *τ* ^2^ was 0.00, *Q* = 0.21, df = 2 and *I* ^2^ was 0% as displayed in Figure 3. Prediction intervals could not be calculated as the estimated *τ* ^2^ was 0.00 (Higgins, 2009).

In addition, a number of secondary outcomes were reported in a single study only. The outcomes were measures on subscales of the CBCL (internalising and externalising subscales), anxiety, loneliness and depression/anxiety diagnosis. The effect sizes and 95% CIs are reported in Table 7.

##### Sensitivity

Sensitivity analyses were planned to evaluate whether the pooled effect sizes were robust across study design and components of methodological quality.

No randomised controlled trials were included in the meta‐analyses, so the impact of study design could not be evaluated. For methodological quality, it was only possible to carry out a sensitivity analysis for the Selection risk of bias item in the meta‐analysis of depressive symptoms. We examined the robustness of our conclusion when we excluded the study with a Moderate risk of bias assessment.

We further examined the robustness of our conclusion to clustered delivery of treatment by reporting results of the meta‐analysis of overall psychosocial adjustment using both an unadjusted effect size and using a substantially higher ICC (0.3) than in the primary analysis.

**Figure 4:**
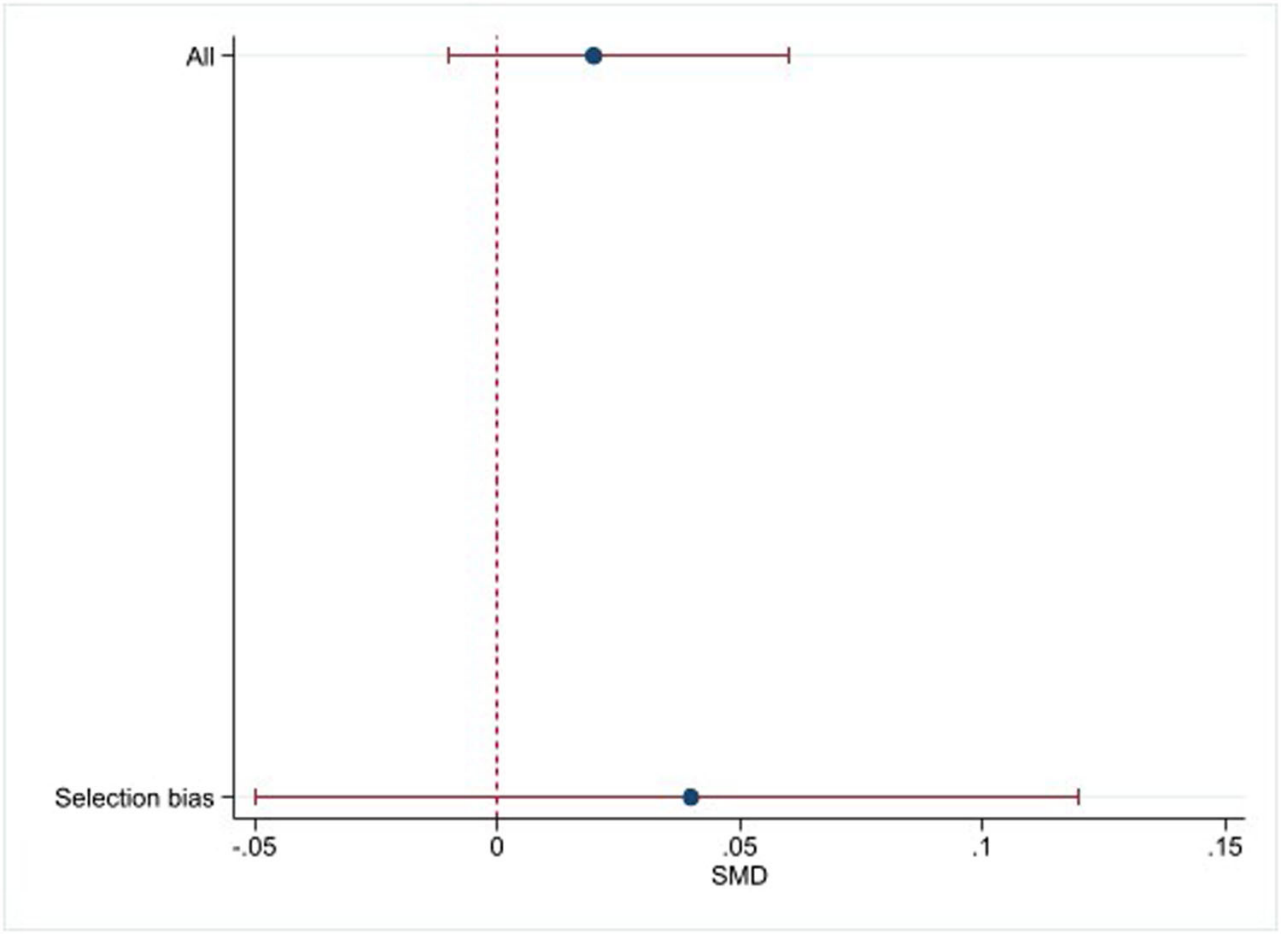
Depressive symptoms Sensitivity.

**Figure 5:**
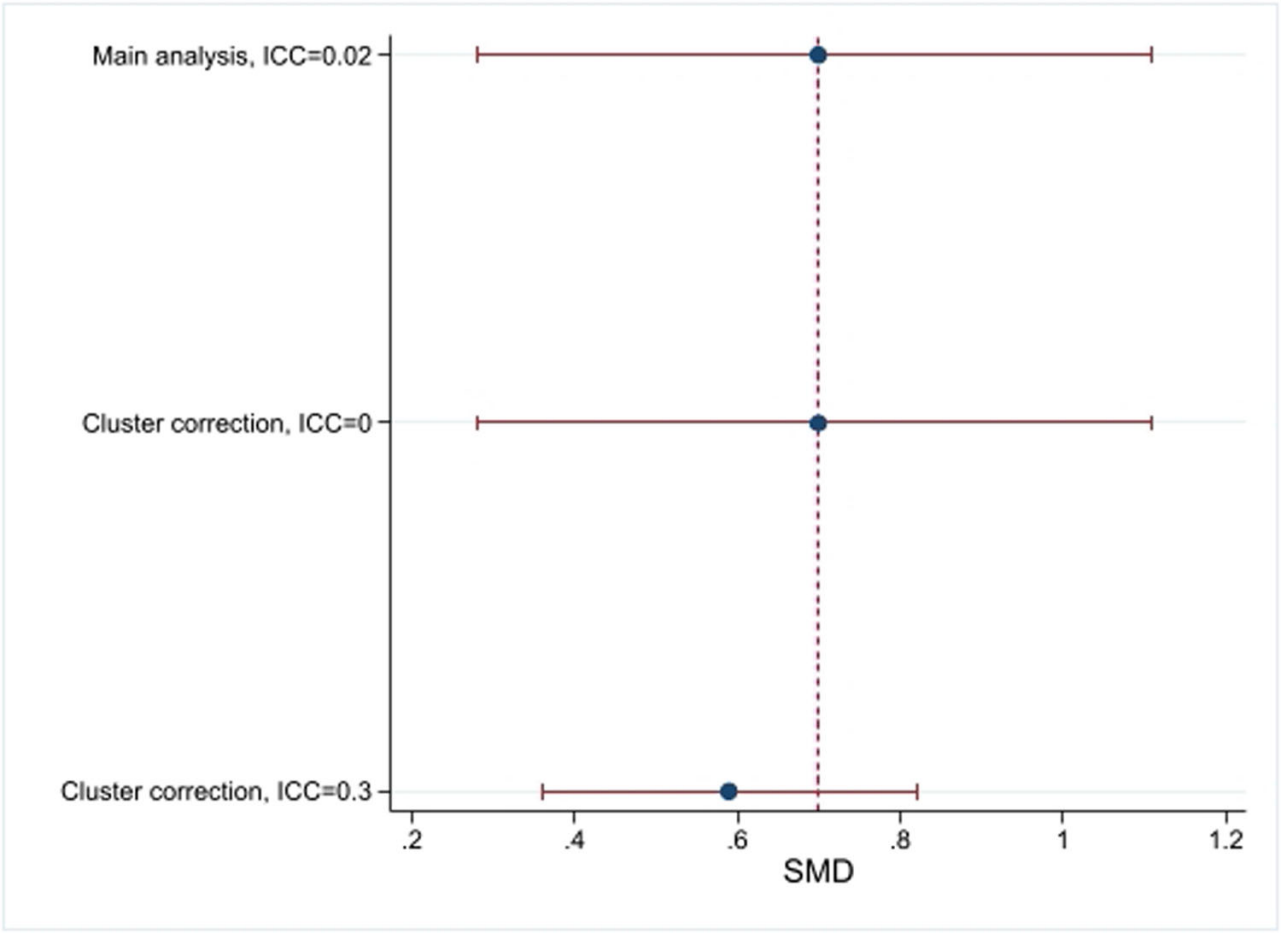
Overall psychosocial adjustment Sensitivity.

The results of the sensitivity analyses are provided in Tables 8 and 9 and displayed in Figures 4 and 5.

**Table 8:** Sensitivity Depressive symptoms.

	Number of studies	ES	95% CI	
Studies excluded	*k*	SMD	Lower	Upper
All	3	0.01	−0.01	0.03
Selection: Moderate risk of bias removed	2	0.04	−0.05	0.12

**Table 9:** Sensitivity Overall psychosocial adjustment.

	Number of studies	ES	95% CI	
	*k*	SMD	Lower	Upper
Main analysis, ICC = 0.02	2	0.70	0.28	1.11
Cluster correction, ICC = 0	2	0.70	0.28	1.11
Cluster correction, ICC = 0.3	2	0.59	0.36	0.82

There were no appreciable changes in the results following removal of any of the studies nor were there any appreciable changes of doubling the effect from the study using time spent in general education as a continuous variable.

In summary, the conclusions of the main synthesis do not change.

##### Publication bias

We were unable to comment on the possibility of publication bias because there were insufficient studies for the construction of funnel plots.

## 6 DISCUSSION

### 6.1 Summary of main results

Overall, there were too few studies included in any of the meta‐analyses in order for us to draw any conclusion concerning the effectiveness of participation in organised sport. At most, the results from three studies could be pooled in a single meta‐analysis. No meta‐analysis was performed on any of the primary outcomes. It was only possible to pool the two secondary outcomes: overall psychosocial adjustment at post intervention and depressive symptoms at 0–3 years follow‐up.

### 6.2 Overall completeness and applicability of evidence

We included in total seven studies in the data synthesis and of these, a maximum of three studies reported the same outcome at a comparable time point and could be used in a specific meta‐analysis. This number is lower than the number of studies (13) meeting the inclusion criteria. The reduction was caused by two different factors.

Five studies were judged to have a Critical risk of bias and, in accordance with the protocol, we excluded these from the data synthesis on the basis that they would be more likely to mislead than inform. Further, one study could not be used in the data synthesis as a Tobit regression model was applied, and the coefficients reported can not be interpreted as the effect of sports participation on the outcomes.

If all the included studies had provided an effect estimate with lower risk of bias (or applied a model from which an effect size could have been calculated), the final list of useable studies in the data synthesis would have been larger, which again would have provided a more robust literature on which to base conclusions.

All studies used in the data synthesis were from Australia, the US and Canada. This narrow geographical coverage is a clear limitation of the review.

It was not possible to perform a meta‐analysis on any of the primary outcomes. This is a clear limitation of the review.

It was not possible to examine the impact of the moderators.

### 6.3 Quality of the evidence

All studies (13) used non‐randomised designs. Overall, the risk of bias in the included studies was high. Five studies were rated Critical risk of bias. The level ‘Critical’ means: the study (outcome) is too problematic in this domain to provide any useful evidence on the effects of intervention, and it is excluded from the data synthesis. The remaining studies were all assessed to have some concerns overall. Six studies were rated Serious risk of bias overall and two studies were rated Moderate risk of bias overall.

We examined the risk of bias using Cochrane's revised risk of bias tool, the model ROBINS–I, developed by members of the Cochrane Bias Methods Group and the Cochrane Non‐Randomised Studies Methods Group (Sterne et al., 2016a) for non‐randomised studies.

The quality of the evidence in this review was enhanced by excluding studies assessed to be at critical risk of bias using the ROBINS–I tool from the data synthesis. We believe this process excluded those studies that are more likely to mislead than inform.

With at most three studies contributing effect sizes for two secondary outcomes (and reported at two different time points), it is of little use to discuss overall consistency in the direction and magnitude of effects and heterogeneity between studies.

### 6.4 Potential biases in the review process

We performed a comprehensive electronic database search, combined with grey literature searching, and hand searching of key journals. All citations were screened in teams by two independent screeners, one review author (TF) and one research assistant (FLWS).

We believe that all the publicly available studies on the effect of participation in organised sport on young people's risk behaviour and personal, emotional and social skills up to the censor date were identified during the review process.

However, three references were not obtained in full text.

We were unable to comment on the possibility of publication bias as at most three studies were included in the same meta‐analysis. Thus, we cannot rule out that there are still some missing studies which were not published or made public.

We believe that there are no other potential biases in the review process as two review authors (TF and MEV) independently coded the included studies. Any disagreements were resolved by discussion. Further, decisions about inclusion of studies were made by the team of screeners (TF and FLWS) and one further review author (MEV). Assessment of study quality and numeric data extraction was made by one review author (TF) and each study was checked by another review author (MEV).

### 6.5 Agreements and disagreements with other studies or reviews

We located one systematic review on sport programmes for at‐risk youth, or as termed by the authors, socially vulnerable youth (Hermens et al., 2017), however there were no restrictions on study design. The participant population was young people aged 10 to 23 who were socially vulnerable. Socially vulnerable is defined as: ‘Socially vulnerable youth represent a broad group, ranging from youth living in areas of low socioeconomic status (SES) to youth receiving residential care or non‐residential counselling. A common denominator is that they face stressors in their everyday life, such as income poverty, poor family management, low housing quality, and peers being involved in problem behaviour’ (p. 408). This definition of ‘socially vulnerable youth’ is in line with our definition of ‘at‐risk youth’. Studies published during 1990 to December 31, 2014 were included. Only studies that reported results on life skill development outcomes were included. As stated above, there were no restrictions on how the studies measured an impact, that is, qualitative studies as well as quantitative studies with or without comparison groups were included and some studies analysed one sport programme versus another sport programme. No meta‐analysis was performed, only a narrative analysis describing the studies and the results as stated in the studies.

We located another systematic review including a broad range of physical activity programmes for participants aged 4–18 years considered to be at‐risk (Lubans et al., 2012). Participants with eating disorders and diagnosed psychiatric conditions were not eligible. The search was conducted on 21st December 2010 in six electronic databases (EMBASE, OVID MEDLINE, PsychINFO, PubMed, Scopus and SPORTDiscus) and conference abstracts, dissertations, theses and articles published in non‐peer reviewed journals were excluded. Quantitative studies with or without comparison groups reporting on social and emotional well‐being outcomes were included. No meta‐analysis was performed, only a narrative analysis describing the studies and the results as stated in the studies.

Further, we located four systematic reviews on sport programmes (two of them including other physical activities as well) that did not restrict participants to be at‐risk youth.

The review by Eime et al. (2013), searched in June 2012 for studies reporting on the mental and/or social health benefits of sports programmes. They explicitly excluded studies or reports that addressed ‘exercise’, ‘physical activity’, ‘physical education’, or ‘recreation’, and not sport. Both quantitative and qualitative studies were included. After reviewing the included studies, it was decided that studies focusing on children and adolescents should be reviewed separately from studies focusing on adults and the review therefore focused on children and adolescents (18 or less). Only a narrative description of the studies was provided.

The review by Spruit et al. (2016a), included all studies examining the effect of physical activity interventions (including sports) on externalising and internalising problems, self‐concept, and academic achievement published before August 2015. Wilderness or adventure programmes, such as rock climbing, camping, backpacking, and hiking as a form of group therapy were excluded. The age range of the eligible samples had to be between 10 and 21 years old with a mean between 11 and 18. Only experimental studies (defined as studies where a treatment group was compared to a comparison group of juveniles who did not participate in a physical activity intervention) were eligible. Finally, populations with physical health issues (except for obesity) were excluded. In total, 57 studies were included, of which 14 addressed sport interventions. A multilevel meta‐analysis for each of the four outcomes was performed showing overall small‐to moderate effects of physical activity interventions on all four outcomes. The moderating effect of whether the physical activity intervention consisted of sports or (aerobic) exercise activities was only analysed. Only the effect on one outcome differed between sport and (aerobic) exercise activities. Larger effects of physical activity interventions on self‐concept were found when the intervention consisted of (aerobic) exercise compared to sports intervention.

The (almost) same author team performed a systematic review with meta‐analysis on all studies addressing the relationship between sports participation and delinquency in juveniles which were published before October 2015 (Spruit et al., 2016b). Studies measuring sports participation combined with other types of activity participation and studies addressing sport interventions were excluded. The eligible age was reported as all studies with a mean between 12 and 18. Another eligibility criterion was that the study had to contain both athlete and non‐athlete samples, and both delinquent and non‐delinquent samples (or samples of the general population of adolescents), which seems a bit odd to base the study eligibility criteria on the presence of the outcome in the samples. It was not required that the studies measured a causal relationship. In total, 51 studies were included. A multi‐level analysis using correlation as the effect size was performed showing overall no correlation between sport participation and juvenile delinquency. The moderating effect of (amongst other things) the type of sports participation (team vs. individual, contact vs. non‐contact and school setting vs. out‐of‐school setting) were analysed. One significant relationship was found; participants in individual sports were more delinquent than non‐participants, whereas no relationship between participation in team sports and delinquency was found.

Finally, the review by Whitley and colleges (Whitley et al., 2019), reviewed the research on sport‐based youth development interventions conducted within the U.S. The evidence of two types of interventions were searched for, a plus‐sport (i.e., sport adapted to maximise developmental objectives) intervention or a sport‐plus (i.e., sport used as a vehicle for development, with precedence on non‐sporting outcomes) intervention, published from 1995 through August 2017. Eligible programmes should be supplied to participants aged 10–24 years and data collected completely/partly in the U.S. Both quantitative and qualitative studies were included. In total, 56 studies were included reporting on ten different interventions, of which two were explicitly targeting youth from schools serving low‐income communities (Playworks) and at‐risk youth in various settings respectively (Doc Wayne). A narrative description of the results for each intervention was provided.

We specifically searched the Cochrane systematic reviews and located one marginally relevant for the current review (Ekeland et al., 2004). The review by Ekeland, 2004, searched in 2002 (month not reported) for studies reporting on exercise interventions for children and young people. The objective of the review was to determine if exercise interventions can improve self‐esteem amongst children and young people. Eligible activities included gross motor, energetic activity, for example, running, swimming, ball games and out‐door play of moderate to high intensity, or strength training. Only randomised controlled trials and quasi‐randomised trials, for example, those that use alternate allocation, date of birth, and so forth, were eligible study designs. Twenty‐three trials were included, of which several included sports activities in the control condition. Three studies (examining physical exercise/sport and strength training) included at‐risk participants (children with learning disabilities [one study] and juvenile delinquents [two studies]), otherwise only healthy children and adolescents were included. A separate meta‐analysis was performed for these three studies, two of them included sport in the control condition.

Besides being up‐to‐date, a major difference between these five systematic reviews and the current review is that we focused on organised sport programmes targeted at‐risk children/youth aged 6 to 18. We only included studies with a control group. All relevant outcome areas were analysed separately in a meta‐analysis taking into consideration the dependencies between effect sizes.

## 7 AUTHORS' CONCLUSIONS

### 7.1 Implications for practice and policy

Healthy after‐school activities for youth may serve as important resources for reducing school failure and youth crime. Leisure time activities such as organised sport may be a very healthy activity as it provides young people with a valued place within a structured peer‐involved activity, and links young people to coaches who are positioned to assume the role of caring adult mentors, which in particular at‐risk youth may be in need of.

Unfortunately, the evidence on participation in organised sport on risk behaviour, personal, emotional and social skills of young people, who either have experienced or are at‐risk of experiencing an adverse outcome was inconclusive because too few studies could be used in the data synthesis (see section *Overall completeness and applicability of evidenc*e). Further, the few studies included in the data synthesis did not report results on the same type of outcome, leaving very few observations to base a conclusion on. No meta‐analysis was performed on any of the primary outcomes, and at most three effect sizes could be pooled on two secondary outcomes; overall psychosocial adjustment (*n* = 2) and depressive symptoms (*n* = 3).

Finally, the majority of the available evidence used in the data synthesis was from either Canada or the USA (one study was from Australia), countries with a less developed welfare state and social security system (i.e., liberal regime countries) than, for example, the Scandinavian countries with comprehensive welfare state institutions (Esping‐Andersen, 1990). Thus, the findings may not be generalisable to other settings and systems outside North America.

Given the limited number of rigorous studies available, it would be natural to consider conducting randomised controlled trials. However, due to the very nature of the intervention, it is voluntary to participate, and it cannot be prescribed, the population of interest can be encouraged to take it up. More young people could probably be encouraged to self‐select into organised sport if the opportunity was immediately available.

Increasing the availability of opportunities in precisely those communities where the adolescents are at highest risk for poor developmental outcomes would prevent transportation issues from being a barrier to attending organised sport. However, even if programmes are available, they are typically not for free but come with a participation fee and equipment costs out of reach for children living in poverty.

In summary though, since there is no evidence that participation in organised sport is either harmful or beneficial for at‐risk children and youth, the provision of sport opportunities, both facilities and affordability, in childhood and adolescence should be considered in light of other strategies to support at‐risk children and youth, in terms of costs and likely benefits.

### 7.2 Implications for research

Further research is required to fully address the effects of participation in organised sport on young people's risk behaviour. Few studies have investigated this issue using appropriate comparison groups. We found no randomised controlled trials and the risk of bias in most of the included non‐randomised studies was high. By excluding from the data synthesis studies judged to be at critical risk of bias, this review aimed at enhancing the quality of the evidence on the effects of participation in organised sport. We believe this process excluded those studies that are more likely to mislead than inform on the true effect sizes.

Seven of the included studies reported on primary outcomes (problem/risk behaviour). Unfortunately, the risk of bias was critical high in four of these studies to be included in the data syntheses and further one study did not report results that enabled us to calculate an effect size with standard error (a Tobit regression model was used). This leaves us with two studies reporting on the primary outcome to use in the data synthesis and, as two distinct outcomes were reported in the two studies (delinquency in one study and substance use in the other), we could not perform a meta analysis on any of the primary outcomes. Likewise, few studies included in the data synthesis reported results on the same type of secondary outcome, leaving very few observations to base a conclusion on.

Five studies were judged critical risk of bias due to confounding. Four studies had access to very few confounders, all with large imbalances, and one study had access to some important confounding factors (pre‐test scores, gender SES and language minority) but, in addition, included a number of bad controls.

Unfortunately, one included study did not provide data that permitted the calculation of an effect size and standard error (applied a Tobit regression model) and could therefore not be used in the data synthesis.

These considerations point to the need for more rigorously conducted studies. Obtaining balance on important confounding factors may be difficult when participants are not randomised, which adds to the importance of statistically controlling for relevant factors.

It would be possible to perform studies in countries with access to administrative data about the participant's leisure time activities, school, and personal characteristics. Such studies from other countries than the USA and Canada would have the potential of making useful contributions to the sport participation effectiveness literature if due consideration is made to the strengths and weaknesses of the studies found in this review. A limitation of such a strategy is that only administrative outcomes such as delinquincey, suspension and school drop‐out can be included, whereas self‐reported outcomes such as substance use can not be included.

## DATA AND ANALYSES

### Comparison 1

#### Overall psychosocial adjustment

**Table:** 

Outcome or subgroup title	No. of studies	No. of participants	Statistical method	Effect size
1.1 Post	2		Std. Mean Difference (IV, Random, 95% CI)	0.70 [0.28, 1.11]

### Comparison 2

#### Depressive symptoms

**Table:** 

Outcome or subgroup title	No. of studies	No. of participants	Statistical method	Effect size
2.1 Follow up	3		Std. Mean Difference (IV, Random, 95% CI)	0.02 [−0.01, 0.06]

## CONTRIBUTIONS OF AUTHORS

- Content: Else Ladekjær, Trine Filges
- Systematic review methods: Trine Filges
- Statistical analysis: Trine Filges, Mette Verner
- Information retrieval: Elizabeth Bengtsen

## DECLARATIONS OF INTEREST

There are no potential conflicts of interest.

### Preliminary timeframe

Approximate date for submission of the systematic review will be no longer than 2 years after protocol approval.

### Plans for updating this review

Trine Filges will be responsible for updating the review and updates can be expected each second year.

## SOURCES OF SUPPORT

### Internal sources

VIVE Campbell, Denmark.

### External sources

None, Other.

## DIFFERENCES BETWEEN PROTOCOL AND REVIEW

We did not calculate prediction intervals as either (or both) there were too few studies or no heterogeneity in the meta‐analyses.

There were insufficient studies for moderator analysis to be performed.

We were unable to comment on the possibility of publication bias because there were insufficient studies for the construction of funnel plots.

Analysis 1.1 and 1.2

**Analysis 1.1:**
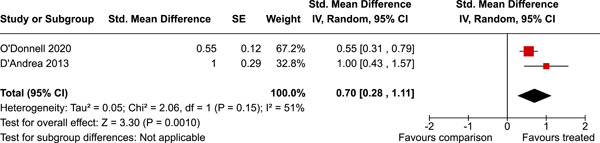
Comparison 1: Overall psychosocial adjustment, Outcome 1: Post.

**Analysis 2.1:**
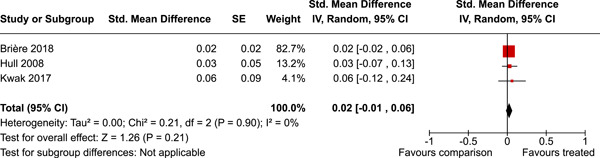
Comparison 2: Depressive symptoms, Outcome 1: Follow up.

### PEER REVIEW

The peer review history for this article is available at https://www.webofscience.com/api/gateway/wos/peer-review/10.1002/cl2.1381.

## Supporting information

Supporting information.

Supporting information.

Supporting information.

Supporting information.

## Data Availability

N/A.
